# 

**Published:** 1913-12-06

**Authors:** 


### Freemasonry in Hospital Life.

HOSPITAL SECRETARY AND MASTER MASON.

A number of persons well known in hospital circles were amongst the officers appointed on November 27 at the installation of Mr. Godfrey Hamilton as Master of the Nosocomia Lodge. The ceremony was performed by the retiring Master, Mr. Stanley Smith, and the other offices were filled as follows: Mr. J. W. Peck, S.W.; Mr. A. Hayward, J.W.; Mr. E. A. Attwood, Treasurer; Mr. R. Kershaw, Secretary; Mr. W. H. Pearce, S.D.; Mr. J. Langford Moore, J.D.; Mr. W. J. Chichele Nourse, D.C.; Mr. F. E. Thorn, Assistant D.C.; Mr. J. McKay, Almoner; Mr. A. E. Dean, Organist; Mr. A. A. Pearce, Assistant Secretary; Mr. W. Pearson, I.G.; Mr. G. A. Arnaudin, Mr. A. H. Coughtrey, and Mr. J. C. Buchanan. Amongst others present at the ceremony were Mr. Perceval A. Nairne, Mr. A. Burkett, Mr. Edgar Penman, and other well-known figures in institutional and Masonic life.

### Appointments.

Mr. G. H. Stevenson, fifth assistant medical officer at Bexley Mental Hospital, has been promoted to be fourth assistant medical officer.

Dr. A. W. Petrie, fourth assistant medical officer at Bexley Mental Hospital, has been appointed third assistant medical officer at Hanwell Mental Hospital, to succeed Mr. W. G. Cheatle, who has left the service.

Reappointments.—Resident medical officer at the Barnes Convalescent Hospital, Cheadle, Cheshire, Mr. G. B. Warburton, M.B., Ch.M. (Vict.), F.R.C.S.; director of the Cancer Research Laboratory, Mr. W. J. Reid, M.A., B.Sc., M.B., Ch.B.

The following appointments have been made at the Hospital for Sick Children, Great Ormond Street: Sir William Arbuthnot Lane, emeritus surgeon; Mr. H. A. T. Fairbank, surgeon to in-patients; and Mr. L. E. Barrington Ward, surgeon to out-patients.

The name of Captain the Hon. Sir Charles Wentworth-Fitzwilliam, K.C.V.O., Master of the Horse, is to be submitted to the next quarterly meeting of the governors of the Leicester Royal Infirmary for election to a life governorship in recognition of donations of £52 10s.

Dr. A. Kenny McKee has resigned the appointment of deputy medical superintendent of the Willesden Council's Isolation Hospital. Owing to the dearth of medical men when the appointment now vacant was advertised recently, the committee expressed the opinion that the salary offered was too small, and decided that the post be now again advertised at a commencing salary of £200, rising by annual increments of £25 to £250.

